# Effect from Autoclave Sterilization and Usage on the Fracture Resistance of Heat-Treated Nickel–Titanium Rotary Files

**DOI:** 10.3390/ma16062261

**Published:** 2023-03-11

**Authors:** Rashid El Abed, Dana Al Raeesi, Aisha Alshehhi, Zuhair Alkhatib, Amar H. Khamis, Mohamed Jamal, Hyeon-Cheol Kim

**Affiliations:** 1 Endodontic Department, Hamdan Bin Mohammed College of Dental Medicine, Mohammed Bin Rashid University of Medicine and Health Sciences, Dubai 505055, United Arab Emirates; 2 Endodontic Department, Emirates Health Services Establishment, Dubai, United Arab Emirates; 3 Emirates Endodontic Society, Dubai, United Arab Emirates; 4 Biostatistics Department, Hamdan Bin Mohammed College of Dental Medicine, Mohammed Bin Rashid University of Medicine and Health Sciences, Dubai 505055, United Arab Emirates; 5 Department of Conservative Dentistry, School of Dentistry, Dental Research Institute, Pusan National University, Yangsan 50612, Republic of Korea; 6 Dental and Life Science Institute, Pusan National University, Yangsan 50612, Republic of Korea

**Keywords:** autoclave sterilization, dynamic fatigue, fracture resistance, heat treatment, nickel–titanium file, torsional strength

## Abstract

This study aimed to assess the effect of mechanical loading and heating on the cyclic fatigue and torsional fracture resistances of heat-treated nickel–titanium files after usage and autoclaving. Sixty files (One Curve) were tested for cyclic fatigue and torsional fracture resistances using customized devices. The files were divided into three groups according to the test conditions (*n* = 10); new (group-N), used for simulated canal shaping (group-U), and sterilized after use (group-S). For cyclic fatigue resistances, the files were freely rotated in a curved metal canal under body temperature; the time elapsed to fracture was recorded and the numbers of cycles to fracture (NCF) were calculated. For the torsional resistances, the file tip was fixed and rotated until the file fractured. The maximum torsional load and distortion angle were recorded. The toughness was calculated. Fracture fragments were examined with a scanning electron microscope. Data were analyzed using a one-way analysis of variance and Tukey’s post hoc test at the significance level of 95%. Group-U showed significantly higher NCF than group-S (*p* < 0.05). However, there was no significant differences between groups-N and -S in the NCF (*p* > 0.05). Group-N showed a significantly bigger distortional angle and higher torsional toughness than groups-U and -S, but the ultimate torsional strength did not have significant difference between the groups. Under the limitation of this study, autoclave sterilization after single-usage did not improve the fracture resistance of heat-treated One Curve nickel–titanium files.

## 1. Introduction

Since the introduction of endodontic nickel–titanium (NiTi) rotary files in the 1980s [1], various efforts have been made to overcome the fracture risk of NiTi files and improve the reliability and efficiency of the rotary instruments [2].

The main concern regarding NiTi files in clinical use is their unexpected fracture due to flexural fatigue and torsion [3]. Cyclic fatigue fracture was reported to occur when repeated tensile and compressive stresses were concentrated on the NiTi instrument during rotating in a curved canal [4], while torsional failure happens when the tip of an instrument, or any part, becomes locked in the canal walls, whereas its shaft rotates continuously, causing a breakage when the force exceeds the alloy’s plastic limit [5].

To overcome the drawbacks of NiTi engine-driven files, investigators have dedicated their attention on the NiTi alloys, design improvements, and new surface treatments such as ion implantation, electropolishing, and heat treatments to improve the mechanical properties and efficiencies [2,6,7]. One of the best methods for modifying the transition temperatures of NiTi alloys and enhancing the fatigue resistance of NiTi rotary instruments is heat treatment (thermomechanical processing) [8]. Evident changes are well-known in the metallurgical properties of NiTi alloys due to the impact of thermomechanical treatment [9].

When stress is applied to the instrument, microstructural changes occur, which in turn cause phase transformation [6]. Not only the stress but also the heat application on the NiTi alloy changes the mechanical properties of the rotary instruments. Cyclic fatigue and/or torsional loading generated during the instrumentation procedures result in increased mechanical properties according to the extent of loaded stresses [5,10]. It was also reported that the additional stress loading and heat application may reduce the fracture resistance [11].

In clinical practice, the NiTi rotary files are usually reused for economic reasons, which subjects them to repeated autoclave sterilization [12,13]. The dilemma of whether endodontic instruments can be used again after being sterilized raises the question of the extent to which these procedures influence their physical and mechanical properties. It was reported that NiTi alloys exhibit higher corrosion rates at higher temperatures [14]. Since thermal treatment plays a crucial role in the NiTi manufacturing processes, the impact of heat-generating sterilization procedures on the mechanical properties of these alloys are of specific concern.

Contrast results have been reported on the probable effects of heat sterilization on NiTi instrument fracture resistance: some findings suggested that sterilization may enhance NiTi torsional strength [15], others showed a lower torsional moment and enhanced cycle fatigue susceptibility [16,17,18,19].

The recently introduced One Curve (MicroMega, Besançon, France) is a single rotary file system that underwent an exclusively developed and implemented heat treatment (C-Wire, as called by the manufacturer) and was subjected to electropolishing [20], which provides the capability of being pre-curved. According to the manufacturer, C-wire technology could improve One Curve’s flexibility and adaptability [21,22].

To our knowledge, the effect from the autoclave sterilization of heat-treated rotary files has not been documented enough yet in the literature, especially for One Curve [13,14,15,16,17,18,19]. Thus, this in vitro study aimed to investigate the effect from usage and/or autoclave sterilization on the cyclic fatigue and torsional fracture resistance in body temperature conditions using the heat-treated One Curve file system.

## 2. Materials and Methods

### 2.1. Simulated Canal Preparation

The selected One Curve NiTi file used in this study has an ISO size 25 noncutting tip and it has a variable cross-section (triple-helix section at the tip and an S-section closer to the shank), a constant 6% taper, and a variable pitch.

G*Power v3.1 (Heinrich Heine, University of Düsseldorf, Düsseldorf, Germany) was used to determine the required sample size for the tests, with a 5% level of significance and a test power of 0.80. Before the experimental tests, the files were inspected using a dental operating microscope (Zeiss Pico; Carl Zeiss MediTec, Dublin, CA, USA). Any instruments that were distorted or faulty were eliminated. A total of 60 files were analyzed and randomly divided for the 2 tests (*n* = 30); cyclic fatigue and torsional fracture resistance tests. Those files were divided into three experimental groups (*n* = 10). The group designation and specimen preparation were performed as described by El Abed et al. [23]. Briefly, for group-N, the new files of One Curve were tested to establish a baseline number of both cyclic fatigue and torsional resistance. In groups-U, files were used to shape the J-shaped resin simulated canals (Dentsply Sirona). These simulated canals had a length of 16.5 mm and a curve of 35 degrees. In order to prepare the resin simulated canals for instrumentation with NiTi files, #10K and #15K stainless-steel files (Mani, Tochigi, Japan) were used initially along with irrigation using 20 mL of normal saline and a 27-gauge needle (Endo-Eze; Ultradent, South Jordan, UT, USA).

After pre-enlarging the simulated canals, X-smart plus endodontic motors (Dentsply Sirona) were used to operate single One Curve NiTi files. After instrumentation, the One Curve files in group-U were tested for both cyclic fatigue and torsional fracture resistance.

The files in group-S were also used for single root canal shaping as in group-U. Afterwards, the files were wiped down with an alcohol sponge to remove any remaining resin particles before being sterilized in an autoclave (Steris Amsco Century autoclave; Steris Co, Mentor, OH, USA) at 132 °C for 30 min. After sterilization, the files were tested for both cyclic fatigue and torsional resistances.

### 2.2. Cyclic Fatigue Resistance Test

**Figure 1:**
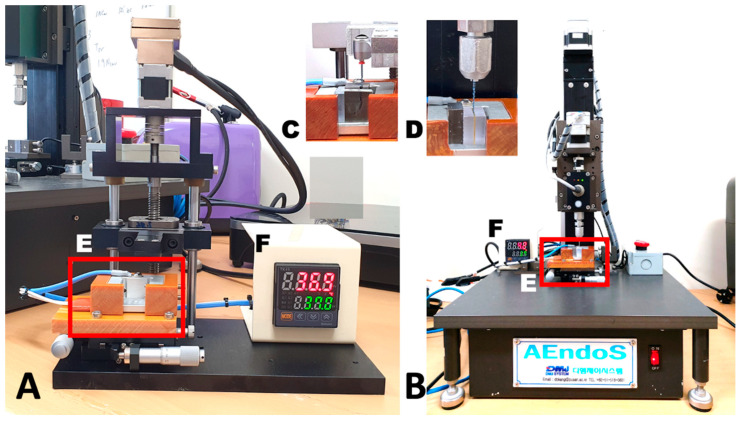
Test devices used in this study. (**A**) Tester (EndoC) for cyclic fatigue resistance test, (**B**) tester (AendoS) for torsional resistance test, (**C**) simulated canal in the heat generation pad, (**D**) Brass plates and polycarbonate blocks holding the file tip, (**E**) heat generation pad, and (**F**) electronic heat controller.

A One Curve file in each of the three groups was rotated with a repetitive up-and-down dynamic mode in a curved canal using a custom-made device (Figure 1A, EndoC; DMJ systems, Busan, Korea). The canal was 17 mm in length, 6 mm in radius, and 35° in curvature (Figure 1C). An electronic heat controller (TK4N/S/SP; Autonics, Busan, Korea) was utilized to transfer heat three-dimensionally and directly to the brass plate where the instrument’s tip was held to mimic body temperature at 37 °C (Figure 1E,F).

The pecking distance comprised a 4 mm increment in each direction every 0.5 s, with a 50 ms dwell time. Using a torque-controlled endodontic motor and handpiece (X-smart Plus), the file was rotated at a constant rate of 300 rpm in the canal. The time to fracture was observed visually and audibly, and the number of cycles to fracture (NCF) was determined using the recorded time to fracture (in seconds). The length of the fractured fragment was measured using a digital microcaliper (Mitutoyo, Kawasaki, Japan).

### 2.3. Experimental Test of Torsional Resistance

The torsional resistance test was performed using a customized device (Figure 1B, AEndoS; DMJ Systems) according to the modified method by Ha et al. [24]. In brief, the file tip was secured between two brass plate blocks three millimeters from the instrument tip. The files were kept straight during the testing, and a clockwise rotation at a constant rotational speed of 2 rpm was applied until the file fractured. The temperature was controlled to simulate the body temperature as 37 °C using the electronic heat controller (Figure 1E,F).

During the rotation of the files, the ultimate strength (Ncm) and the fracture angle (degree, °) were recorded at a rate of 50 Hz using customized software. Using Origin v6.0 Professional, the toughness (°•Ncm) till fracture was calculated from the area under the plot displaying a distortion angle (X-axis) and torsional load (Y-axis) (Microcal Software Inc., Northampton, MA, USA).

### 2.4. Statistical Analysis

To evaluate the data and compare the groups, SPSS (version 25.0; SPSS Inc., Chicago, IL, USA) was used. The Kolmogorov–Smirnov test was used to evaluate the normality of the data. All the parameters tested which had a normal distribution were analyzed using a one-way analysis of variance and Tukey’s post hoc comparison test, except for the distortion angle under torsion that was analyzed using the Kruskal–Wallis test. The significance level was set at 95%.

### 2.5. Scanning Electron Microscopy (SEM) Evaluation

After cyclic fatigue and torsional resistance tests were completed, using fractured specimens, the topography of the fractured fragments was analyzed using a scanning electron microscope (SU8220; Hitachi High Technologies, Tokyo, Japan).

## 3. Results

Under the three different test conditions, the cyclic fatigue resistance and torsional resistance at 3 mm of One Curve files are presented in Table 1.

The files of group-U (used) showed a significantly higher NCF than the sterilized files after use in group-S (*p* < 0.05). However, there was no significant differences between groups-N (new) and -S in the NCF (*p* > 0.05). The new files in group-N showed a significantly bigger distortional angle and higher torsional toughness than group-U and group-S (*p* < 0.05), but the ultimate torsional strength did not have a significant difference between the groups (*p* > 0.05).

**Figure 2:**
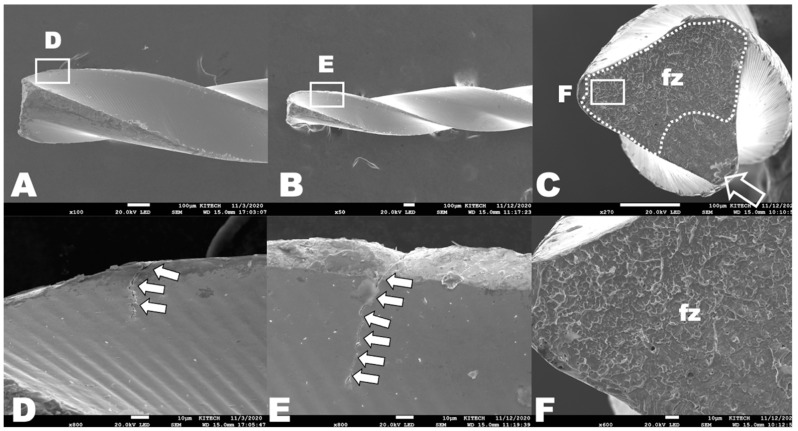
Scanning electron micrographs of the fracture fragments after cyclic fatigue tests. Longitudinal aspects from the samples of group-N (**A**) and group-U (**B**). Cross-sectional aspect from one sample of group-S (**C**,**F**). (**A**,**B**) Fractured instruments from the cyclic fatigue tests did not show macroscopic changes. (**C**) White open arrow indicates crack initiation area. Areas outlined with dot line indicate fatigue fracture zone (fz) with numerous ductile dimples (**F**). (**D**,**E**) White arrows indicate microcracks near the fracture area. No specific differences according to the groups.

**Figure 3:**
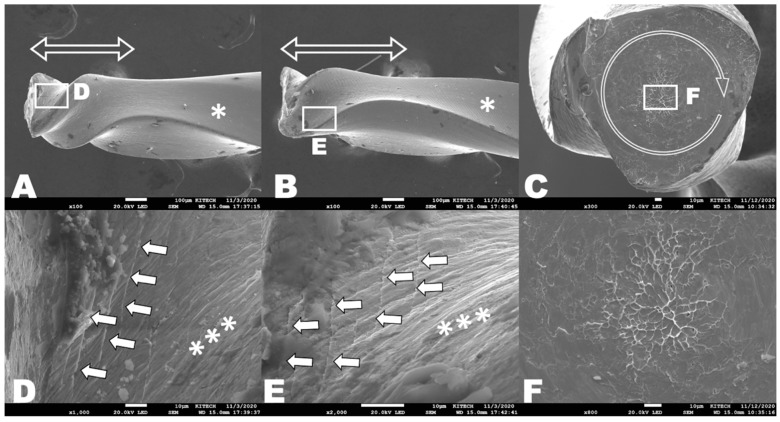
Scanning electron micrographs of the fracture fragments after torsional resistance tests. Longitudinal aspects of the fracture fragments from samples of group-N (**A**) and group-U (**B**). Cross-sectional aspect from one sample of group-S (**C**,**F**). (**A**,**B**) Vacant double-sided arrows indicate the unwound helix of the flute. Specimen of B shows longer unwound helix than the specimen of A. (**C**) Circular arrow shows circular abrasion mark and multiple fibrous dimples (**C**,**F**) at the center of rotation. Linear and straight machining grooves were found at (**A**,**B**) uncompressed area (*) but (**D**,**E**) compressed area shows irregular wrinkles (***) and slippages by microcracks (arrows). No specific differences according to the groups.

The SEM topography analysis revealed the typical appearances of cyclic and torsional failure modes while the test conditions of the three groups in terms of the usage and sterilization did not show any significant differences (Figure 2 and Figure 3).

On cross-sectional views, the cyclic fatigue test specimens had typical topographic features, such as crack initiation areas and a fatigue fracture zone in the cross-sections (Figure 2C,F). Microcracks were discovered near the fracture area from the lateral aspects (Figure 2D,E). The specimens from the torsional resistance tests showed the unwound helix of the flute from the lateral aspects (Figure 3A,B) and had typical appearances, such as circular abrasion marks and skewed dimples near the center of rotation (Figure 3C,F).

## 4. Discussion

Instruments breakage in a root canal usually made problem not only for the prognosis but also for the relationship between patients and dentists. Therefore, after introducing new file systems, researchers often attempt to evaluate their mechanical properties, including the fracture resistances, and compare their efficiencies. Newly developed endodontic rotary files with advanced technology must be assessed in terms of their mechanical properties to provide a reliable recommendation to the endodontist.

The NiTi alloys exists in some crystallographic phases such as austenite, martensite, and R-phase [2,6]. NiTi endodontic file systems may contain either or both austenitic and martensitic crystal structures, and the alloy ratios are varied according to the heat treatments during manufacturing [6,7]. The ratio of alloy composition and phases could be affected by the stress loading and heat applications.

Clinically relevant test methods and scenario are important to increase clinical implications. The present study assessed the effects from the stress loading by the instrumentation procedure and heat application by the sterilization procedures on the files.

There is no international standard for measuring the cyclic fatigue fracture resistance of the NiTi files in experiments. However, several independent variables related to laboratory test conditions or an instrument’s design have been studied to understand their influence on cyclic fatigue strength [25]. Therefore, in this investigation, the cyclic fatigue resistance of One Curve files was tested at body temperature in a dynamic movement with a continuous rotation, which is more relevant to a clinical situation than static rotation.

Interestingly, the NCF of used files (group-U) demonstrated a higher resistance to cyclic fatigue. The potential explanation of the finding could be related to the metallurgic features of the tested instruments, which coincide with the previous studies’ findings [26,27]. During the instrumentation of the simulated canal, the file would have some torsional loading and it could increase the fatigue resistance [5]. Cheung et al. [5] reported that the torsional preloads of the NiTi rotary files within their superelastic limit may improve the cyclic fatigue resistance of the instruments. However, in the present study, heat sterilization of the used file (Group-S) seemed to lower the fatigue resistance of the files, and this was clinically important for a potential reusage. Although cyclic fatigue resistance to NiTi rotary files appears to be improved by torsional preloading [5,11], it was intriguing that the files following autoclave sterilization cycles had reduced mechanical behaviors to fractures.

Presently tested One Curve files are electropolished during the manufacturing process, which, according to the manufacturer, aids in eliminating surface imperfections that weaken the file [20,28]. Although electropolishing appears to increase fatigue strength [29], it has also been reported that electropolishing significantly reduces resistance to cyclic fatigue but has no effect on the torsional resistance [30], which was also noticed in this study.

Unlike cyclic fatigue, the torsional resistance model was standardized by the American National Standards Institute of the American Dental Association, with further specifications for testing NiTi files [31]. In the present study, although the maximum or ultimate torsional loads at failure for all groups did not differ significantly, the groups-U and -S had significantly lower degrees of rotational distortion. Usually, the outcome of torsional resistance or strength could be highly dependent on the geometric feature of the file [32]. The usage of instrumentation may have unwinding and distortion (Figure 3A,B), which could result in changes in the cross-section or helical angle, which consequently seemed to reduce the angle or rotation at fracture.

When NiTi files were subjected to autoclave sterilization, it was observed that titanium oxides increased and the nickel surface concentrations decreased, indicating that surface oxidation may be occurring [33,34]. According to some studies, autoclaving increased torsional strength [15]; other studies showed a loss of strength [18]. Additionally, the torsional fracture resistance of NiTi files appears to be affected by cyclic fatigue preloading, which merits further investigation after autoclave sterilization cycles [10].

In this study, only one heat-treated instruments made of C-wire were tested. The heat application or stress loading on other file systems with a different heat treatment may result in a different metallurgic change and mechanical properties. Therefore, using various file systems made of different heat-treated alloys should be tested with the presented test conditions.

This study included the clinically implacable conditions of reuse after sterilization, but it was only one cycle of heat application. Considering that more than one cycle of reuse after sterilization would happen in clinic, an evaluation of other groups with a few numbers of the reuse and sterilization time would be required. Thus, further research on multiple autoclaving cycles and their influence on the mechanical properties of various heat-treated NiTi file systems is recommended.

## 5. Conclusions

Within the limitations of this in vitro study, the instrumentation load and/or autoclave sterilization of heat-treated nickel–titanium files may change their fracture resistances. Single time usage may retain similar fracture resistances of the One Curve rotary files, except the torsional toughness, but autoclave sterilization after usage may have a reduced cyclic fatigue resistance than the used file before sterilization. The potential benefits or limitations associated with autoclaving previously used files shall be considered and clinicians must be cautioned regarding the reuse of a file after sterilization.

## Figures and Tables

**Table 1:** Torsional and cyclic fatigue resistance of the tested NiTi files.

	Torsional Resistance			Cyclic Fatigue Resistance	
	Ultimate Strength (Ncm)	Distortion Angle (°)	Toughness (°•Ncm)	NCF	Fragment Length (mm)
Group-N	0.70 ± 0.10 ^a^	548 ± 40 ^a^	301.82 ± 56.36 ^a^	1382 ± 129 ^ab^	2.74 ± 0.31
Group-U	0.64 ± 0.05 ^a^	465 ± 48 ^b^	216.13 ± 37.99 ^b^	1527 ± 136 ^a^	2.68 ± 0.23
Group-S	0.66 ± 0.05 ^a^	459 ± 47 ^b^	216.61 ± 35.11 ^b^	1286 ± 219 ^b^	2.97 ± 0.41
*p*-value	0.488	<0.001	<0.001	0.012	0.130

NCF, number of cycles to failure. Group-N, new files; group-U, files underwent canal instrumentation in a simulated J-shaped endodontic block; and group-S, files underwent canal instrumentation and followed by autoclave sterilization. ^a,b^; Values with different superscripts letters are significantly different among the groups (*p* < 0.05). The distortion angle did not have normal distribution and the data were analyzed using a nonparametric Kruskal–Wallis test (*p* < 0.05).

## Data Availability

The data presented in this study are available on request from the corresponding author.
